# *catena*-Poly[[[2-(2-pyrid­yl)-1*H*-benz­imidazole]cadmium(II)]-μ-benzene-1,4-dicarboxyl­ato]

**DOI:** 10.1107/S1600536809026890

**Published:** 2009-07-15

**Authors:** Hai-Yan Liu, Da-Wei Zhao, Hong-Mei Sun

**Affiliations:** a Department of Chemistry and Pharmaceutical Engineering, Suihua University, Suihua 152061, People’s Republic of China

## Abstract

In the title compound, [Cd(C_8_H_4_O_4_)(C_12_H_9_N_3_)]_*n*_, each Cd^II^ ion is six-coordinated in a distorted octa­hedral geometry by four carboxyl­ate O atoms from two benzene-1,4-dicarboxyl­ate anions (*L*), and two N atoms from one 2-(2-pyrid­yl)benzimidazole ligand. The neighboring Cd^II^ ions are bridged by the *L* ligands, forming a zigzag polymeric chain structure. The chains are further extended into a three-dimensional supra­molecular structure through inter­molecular N—H⋯O hydrogen bonds.

## Related literature

For metal-dicarboxyl­ate complexes with aromatic *N*-donor chelating ligands, see: Robl (1992 ▶); Wang *et al.* (2006 ▶); Liu *et al.* (2008 ▶); Xia *et al.* (2007 ▶). For the synthesis, see: Addison & Burke (1981 ▶).
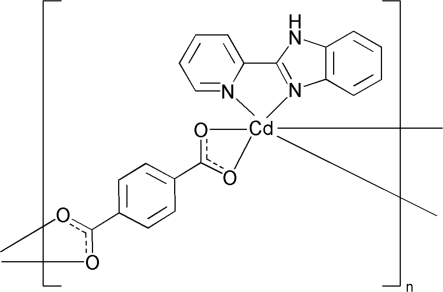


## Experimental

### 

#### Crystal data

[Cd(C_8_H_4_O_4_)(C_12_H_9_N_3_)]
*M* *_r_* = 471.73
Monoclinic, 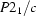

*a* = 7.378 (5) Å
*b* = 20.860 (5) Å
*c* = 11.546 (5) Å
β = 93.362 (5)°
*V* = 1773.9 (15) Å^3^
*Z* = 4
Mo *K*α radiation
μ = 1.26 mm^−1^
*T* = 293 K
0.24 × 0.20 × 0.16 mm

#### Data collection

Oxford Diffraction Gemini R Ultra diffractometer
Absorption correction: multi-scan (*CrysAlis RED*; Oxford Diffraction, 2006 ▶) *T*_min_ = 0.750, *T*_max_ = 0.815
8112 measured reflections
3624 independent reflections
1967 reflections with *I* > 2σ(*I*)
*R*_int_ = 0.052

#### Refinement

*R*[*F* ^2^ > 2σ(*F* ^2^)] = 0.037
*wR*(*F* ^2^) = 0.052
*S* = 0.76
3624 reflections
257 parameters
1 restraint
H atoms treated by a mixture of independent and constrained refinement
Δρ_max_ = 0.47 e Å^−3^
Δρ_min_ = −0.39 e Å^−3^

### 

Data collection: *CrysAlis CCD* (Oxford Diffraction, 2006 ▶); cell refinement: *CrysAlis CCD*; data reduction: *CrysAlis RED* (Oxford Diffraction, 2006 ▶); program(s) used to solve structure: *SHELXS97* (Sheldrick, 2008 ▶); program(s) used to refine structure: *SHELXL97* (Sheldrick, 2008 ▶); molecular graphics: *SHELXTL-Plus* (Sheldrick, 2008 ▶); software used to prepare material for publication: *SHELXL97*.

## Supplementary Material

Crystal structure: contains datablocks global, I. DOI: 10.1107/S1600536809026890/ci2840sup1.cif

Structure factors: contains datablocks I. DOI: 10.1107/S1600536809026890/ci2840Isup2.hkl

Additional supplementary materials:  crystallographic information; 3D view; checkCIF report

## Figures and Tables

**Table 1:** Selected bond lengths (Å)

Cd1—O3^i^	2.271 (3)
Cd1—N1	2.278 (3)
Cd1—O2	2.318 (3)
Cd1—N2	2.322 (3)
Cd1—O1	2.338 (3)
Cd1—O4^i^	2.357 (3)

Symmetry code: (i) 
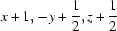
.

**Table 2:** Hydrogen-bond geometry (Å, °)

*D*—H⋯*A*	*D*—H	H⋯*A*	*D*⋯*A*	*D*—H⋯*A*
N3—H1*A*⋯O2^ii^	0.83 (2)	2.17 (3)	2.882 (5)	144 (4)
N3—H1*A*⋯O3^iii^	0.83 (2)	2.46 (4)	2.988 (5)	123 (4)

Symmetry codes: (ii) 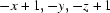; (iii) 
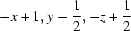
.

**Fig. 1.**
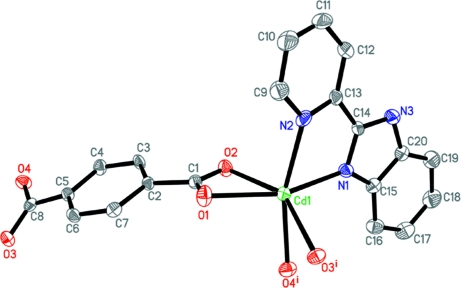
The coordination environment of the CdII ion in the title compound, showing the atom-numbering scheme. Displacement ellipsoids are drawn at the 30% probability level. Symmetry code: (i) 1 + x, 1/2 -y, 1/2 + z.

**Fig. 2.**
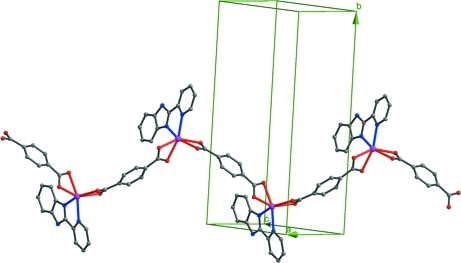
Part of the polymeric chain in the title compound.

## supplementary crystallographic information

### Comment

In recent years, studies on metal-dicarboxylate complexes with aromatic N-donor chelating ligands have attracted special attention because of their interesting structural and chemical properties (Robl, 1992; Wang *et al.*, 2006; Liu *et al.*, 2008; Xia *et al.*, 2007). Herein, we present a new cadmium-dicarboxylate complex (I), namely, [Cd(PyBM)*L*]_n_, where PyBM is 2-(2-pyridyl)benzimidazole and *L* is benzene-1,4-dicarboxylic acid.

Selected bond distances are listed in Table 1. Each Cd^II^ center is six-coordinated by two N atoms of the chelating PyBM ligand and four O atoms from two L ions. The neighboring Cd^II^ ions are bridged by *L* ligands to form a zigzag polymeric chain structure (Fig. 2).

In the crystal structure, the adjacent chains are linked via N—H···O hydrogen bonds (Table 2) resulting in the formation of a three-dimensional supramolecular structure.

### Experimental

2-(2-Pyridyl)benzimidazole was synthesized according to the literature method of Addison *et al.*, (1981). A solution of Cd(CH_3_COO)_2_.2H_2_O (0.133 g, 0.5 mmol), 2-(2-pyridyl)benzimidazole (0.097 g, 0.5 mmol), benzene-1,4-dicarboxylic acid (0.083 g, 0.5 mmol) in H_2_O (10 ml) and CH_3_OH (5 ml) was stirred under ambient conditions, then sealed in a Teflon-lined steel vessel, heated at 443 K for 3 d, and cooled to room temperature. The resulting product was recovered by filtration, washed with distilled water and dried in air (65% yield).

### Refinement

The H atom bonded to atom N3 was located in a difference map and refined with the N-H distance restrained to 0.85 (2) Å. C-bound H atoms were positioned geometrically (C-H = 0.93 Å) and refined as riding, with *U*_iso_(H) = 1.2*U*_eq_(C).

### Crystal data

**Table:**

[Cd(C_8_H_4_O_4_)(C_12_H_9_N_3_)]	*F*(000) = 936
*M**_r_* = 471.73	*D*_x_ = 1.766 Mg m^−^^3^
Monoclinic, *P*2_1_/*c*	Mo *K*α radiation, λ = 0.71069 Å
Hall symbol: -P 2ybc	Cell parameters from 3624 reflections
*a* = 7.378 (5) Å	θ = 2.0–26.5°
*b* = 20.860 (5) Å	µ = 1.26 mm^−^^1^
*c* = 11.546 (5) Å	*T* = 293 K
β = 93.362 (5)°	Block, colourless
*V* = 1773.9 (15) Å^3^	0.24 × 0.20 × 0.16 mm
*Z* = 4	

### Data collection

**Table:**

Oxford Diffraction Gemini R Ultra diffractometer	3624 independent reflections
Radiation source: fine-focus sealed tube	1967 reflections with *I* > 2σ(*I*)
graphite	*R*_int_ = 0.052
Detector resolution: 10.0 pixels mm^-1^	θ_max_ = 26.5°, θ_min_ = 2.0°
ω scans	*h* = −9→9
Absorption correction: multi-scan (*CrysAlis RED*; Oxford Diffraction, 2006)	*k* = −20→25
*T*_min_ = 0.750, *T*_max_ = 0.815	*l* = −9→14
8112 measured reflections	

### Refinement

**Table:**

Refinement on *F*^2^	Primary atom site location: structure-invariant direct methods
Least-squares matrix: full	Secondary atom site location: difference Fourier map
*R*[*F*^2^ > 2σ(*F*^2^)] = 0.037	Hydrogen site location: inferred from neighbouring sites
*wR*(*F*^2^) = 0.052	H atoms treated by a mixture of independent and constrained refinement
*S* = 0.76	*w* = 1/[σ^2^(*F*_o_^2^) + (0.0138*P*)^2^] where *P* = (*F*_o_^2^ + 2*F*_c_^2^)/3
3624 reflections	(Δ/σ)_max_ = 0.001
257 parameters	Δρ_max_ = 0.47 e Å^−^^3^
1 restraint	Δρ_min_ = −0.39 e Å^−^^3^

### Special details

**Table:**

Geometry. All e.s.d.'s (except the e.s.d. in the dihedral angle between two l.s. planes) are estimated using the full covariance matrix. The cell e.s.d.'s are taken into account individually in the estimation of e.s.d.'s in distances, angles and torsion angles; correlations between e.s.d.'s in cell parameters are only used when they are defined by crystal symmetry. An approximate (isotropic) treatment of cell e.s.d.'s is used for estimating e.s.d.'s involving l.s. planes.
Refinement. Refinement of *F*^2^ against ALL reflections. The weighted *R*-factor *wR* and goodness of fit *S* are based on *F*^2^, conventional *R*-factors *R* are based on *F*, with *F* set to zero for negative *F*^2^. The threshold expression of *F*^2^ > σ(*F*^2^) is used only for calculating *R*-factors(gt) *etc*. and is not relevant to the choice of reflections for refinement. *R*-factors based on *F*^2^ are statistically about twice as large as those based on *F*, and *R*- factors based on ALL data will be even larger.

### Fractional atomic coordinates and isotropic or equivalent isotropic displacement parameters (Å^2^)

**Table:**

	*x*	*y*	*z*	*U*_iso_*/*U*_eq_	
Cd1	0.79275 (4)	0.109528 (16)	0.34633 (3)	0.03417 (10)	
C1	0.5456 (5)	0.14696 (17)	0.1841 (4)	0.0286 (10)	
C2	0.4090 (5)	0.17967 (17)	0.1042 (3)	0.0256 (9)	
C3	0.2232 (5)	0.17395 (19)	0.1229 (3)	0.0330 (11)	
H3	0.1850	0.1461	0.1795	0.040*	
C4	0.0980 (5)	0.20935 (18)	0.0576 (3)	0.0341 (10)	
H4	−0.0251	0.2041	0.0685	0.041*	
C5	0.1527 (5)	0.25280 (17)	−0.0243 (4)	0.0293 (10)	
C6	0.3364 (5)	0.25629 (17)	−0.0456 (4)	0.0316 (10)	
H6	0.3742	0.2834	−0.1034	0.038*	
C7	0.4622 (5)	0.22008 (18)	0.0176 (3)	0.0318 (10)	
H7	0.5842	0.2228	0.0020	0.038*	
C8	0.0192 (5)	0.29813 (19)	−0.0795 (3)	0.0302 (10)	
C9	0.7918 (6)	−0.0301 (2)	0.2117 (4)	0.0487 (13)	
H9	0.8198	−0.0058	0.1477	0.058*	
C10	0.7574 (6)	−0.0939 (2)	0.1950 (4)	0.0581 (14)	
H10	0.7656	−0.1127	0.1225	0.070*	
C11	0.7105 (6)	−0.1293 (2)	0.2886 (5)	0.0556 (14)	
H11	0.6836	−0.1726	0.2800	0.067*	
C12	0.7032 (5)	−0.1004 (2)	0.3951 (4)	0.0451 (11)	
H12	0.6712	−0.1238	0.4593	0.054*	
C13	0.7440 (5)	−0.03626 (19)	0.4051 (4)	0.0327 (10)	
C14	0.7435 (5)	−0.00048 (19)	0.5139 (4)	0.0303 (10)	
C15	0.7579 (5)	0.0770 (2)	0.6369 (4)	0.0333 (11)	
C16	0.7781 (5)	0.1363 (2)	0.6920 (4)	0.0436 (12)	
H16	0.7981	0.1737	0.6507	0.052*	
C17	0.7668 (6)	0.1367 (2)	0.8101 (4)	0.0506 (14)	
H17	0.7789	0.1756	0.8493	0.061*	
C18	0.7379 (6)	0.0815 (2)	0.8736 (4)	0.0582 (14)	
H18	0.7312	0.0842	0.9537	0.070*	
C19	0.7192 (6)	0.0225 (2)	0.8197 (4)	0.0490 (13)	
H19	0.6998	−0.0148	0.8613	0.059*	
C20	0.7307 (5)	0.02185 (19)	0.7012 (4)	0.0339 (11)	
N1	0.7647 (4)	0.06143 (15)	0.5215 (3)	0.0329 (8)	
N2	0.7879 (4)	−0.00038 (16)	0.3145 (3)	0.0378 (9)	
N3	0.7210 (5)	−0.02713 (18)	0.6197 (3)	0.0400 (10)	
O1	0.7077 (4)	0.14386 (12)	0.1581 (3)	0.0435 (7)	
O2	0.4940 (3)	0.12531 (12)	0.2787 (2)	0.0371 (8)	
O3	0.0764 (3)	0.34878 (12)	−0.1266 (2)	0.0354 (7)	
O4	−0.1483 (3)	0.28802 (12)	−0.0740 (2)	0.0403 (8)	
H1A	0.701 (6)	−0.0642 (12)	0.642 (4)	0.080 (18)*	

### Atomic displacement parameters (Å^2^)

**Table:**

	*U*^11^	*U*^22^	*U*^33^	*U*^12^	*U*^13^	*U*^23^
Cd1	0.03531 (15)	0.03201 (15)	0.03438 (19)	−0.00389 (19)	−0.00479 (12)	0.0038 (2)
C1	0.035 (2)	0.024 (2)	0.026 (3)	0.0038 (19)	−0.002 (2)	−0.0041 (19)
C2	0.031 (2)	0.024 (2)	0.022 (3)	0.0032 (18)	0.0023 (18)	0.0007 (19)
C3	0.034 (2)	0.036 (2)	0.029 (3)	−0.004 (2)	0.004 (2)	0.013 (2)
C4	0.028 (2)	0.045 (3)	0.029 (3)	−0.001 (2)	0.002 (2)	0.005 (2)
C5	0.032 (2)	0.025 (2)	0.031 (3)	0.0029 (19)	−0.0011 (19)	0.0009 (19)
C6	0.037 (2)	0.032 (2)	0.027 (3)	−0.004 (2)	0.006 (2)	0.004 (2)
C7	0.027 (2)	0.041 (3)	0.028 (3)	−0.004 (2)	0.005 (2)	−0.003 (2)
C8	0.036 (3)	0.033 (2)	0.021 (3)	0.000 (2)	−0.001 (2)	−0.008 (2)
C9	0.057 (3)	0.044 (3)	0.045 (4)	0.005 (2)	0.002 (2)	−0.004 (3)
C10	0.072 (3)	0.054 (4)	0.047 (4)	0.015 (3)	−0.004 (3)	−0.021 (3)
C11	0.062 (3)	0.036 (3)	0.067 (4)	0.007 (2)	−0.013 (3)	−0.020 (3)
C12	0.052 (3)	0.032 (3)	0.050 (3)	0.005 (2)	−0.005 (2)	0.001 (2)
C13	0.034 (2)	0.030 (3)	0.033 (3)	0.011 (2)	−0.005 (2)	−0.002 (2)
C14	0.026 (2)	0.030 (3)	0.034 (3)	0.0012 (19)	−0.003 (2)	0.005 (2)
C15	0.026 (2)	0.039 (3)	0.036 (3)	0.003 (2)	0.005 (2)	−0.003 (2)
C16	0.050 (3)	0.038 (3)	0.044 (4)	−0.003 (2)	0.008 (2)	−0.008 (2)
C17	0.052 (3)	0.058 (3)	0.043 (4)	−0.002 (3)	0.010 (3)	−0.020 (3)
C18	0.070 (4)	0.071 (4)	0.034 (4)	−0.004 (3)	0.007 (3)	−0.013 (3)
C19	0.055 (3)	0.060 (3)	0.032 (3)	−0.005 (2)	0.007 (2)	0.008 (3)
C20	0.031 (2)	0.037 (3)	0.034 (3)	0.003 (2)	0.002 (2)	−0.003 (2)
N1	0.041 (2)	0.028 (2)	0.029 (3)	−0.0010 (17)	0.0010 (17)	−0.0043 (17)
N2	0.046 (2)	0.041 (2)	0.025 (3)	−0.0010 (17)	−0.0062 (19)	−0.0089 (19)
N3	0.047 (2)	0.039 (2)	0.033 (3)	−0.003 (2)	0.001 (2)	0.004 (2)
O1	0.0297 (14)	0.0664 (18)	0.0346 (19)	0.0045 (16)	0.0043 (13)	0.0090 (17)
O2	0.0307 (14)	0.046 (2)	0.035 (2)	−0.0055 (13)	0.0037 (14)	0.0123 (15)
O3	0.0336 (16)	0.0359 (17)	0.037 (2)	0.0027 (13)	0.0036 (14)	0.0105 (14)
O4	0.0291 (16)	0.0356 (17)	0.056 (2)	−0.0032 (14)	−0.0015 (15)	0.0059 (15)

### Geometric parameters (Å, °)

**Table:**

Cd1—O3^i^	2.271 (3)	C9—H9	0.9300
Cd1—N1	2.278 (3)	C10—C11	1.369 (6)
Cd1—O2	2.318 (3)	C10—H10	0.9300
Cd1—N2	2.322 (3)	C11—C12	1.374 (6)
Cd1—O1	2.338 (3)	C11—H11	0.9300
Cd1—O4^i^	2.357 (3)	C12—C13	1.375 (5)
Cd1—C1	2.654 (4)	C12—H12	0.9300
Cd1—C8^i^	2.658 (4)	C13—N2	1.341 (5)
C1—O1	1.252 (4)	C13—C14	1.461 (6)
C1—O2	1.261 (4)	C14—N1	1.303 (4)
C1—C2	1.491 (5)	C14—N3	1.361 (5)
C2—C7	1.382 (5)	C15—N1	1.375 (5)
C2—C3	1.405 (5)	C15—C20	1.389 (5)
C3—C4	1.373 (5)	C15—C16	1.396 (5)
C3—H3	0.9300	C16—C17	1.372 (6)
C4—C5	1.387 (5)	C16—H16	0.9300
C4—H4	0.9300	C17—C18	1.389 (6)
C5—C6	1.394 (5)	C17—H17	0.9300
C5—C8	1.482 (5)	C18—C19	1.383 (6)
C6—C7	1.373 (5)	C18—H18	0.9300
C6—H6	0.9300	C19—C20	1.376 (6)
C7—H7	0.9300	C19—H19	0.9300
C8—O4	1.259 (4)	C20—N3	1.387 (5)
C8—O3	1.272 (4)	N3—H1A	0.829 (19)
C8—Cd1^ii^	2.658 (4)	O3—Cd1^ii^	2.271 (3)
C9—N2	1.342 (5)	O4—Cd1^ii^	2.357 (3)
C9—C10	1.366 (5)		
			
O3^i^—Cd1—N1	100.24 (11)	O3—C8—C5	119.0 (3)
O3^i^—Cd1—O2	147.38 (10)	O4—C8—Cd1^ii^	62.44 (19)
N1—Cd1—O2	103.14 (10)	O3—C8—Cd1^ii^	58.55 (19)
O3^i^—Cd1—N2	113.93 (11)	C5—C8—Cd1^ii^	171.9 (3)
N1—Cd1—N2	72.79 (12)	N2—C9—C10	124.2 (5)
O2—Cd1—N2	94.67 (10)	N2—C9—H9	117.9
O3^i^—Cd1—O1	101.85 (10)	C10—C9—H9	117.9
N1—Cd1—O1	157.89 (10)	C9—C10—C11	117.9 (5)
O2—Cd1—O1	56.34 (9)	C9—C10—H10	121.1
N2—Cd1—O1	98.89 (11)	C11—C10—H10	121.1
O3^i^—Cd1—O4^i^	56.74 (9)	C10—C11—C12	119.7 (4)
N1—Cd1—O4^i^	94.44 (11)	C10—C11—H11	120.2
O2—Cd1—O4^i^	98.78 (9)	C12—C11—H11	120.2
N2—Cd1—O4^i^	163.31 (11)	C11—C12—C13	118.8 (4)
O1—Cd1—O4^i^	96.78 (10)	C11—C12—H12	120.6
O3^i^—Cd1—C1	125.02 (11)	C13—C12—H12	120.6
N1—Cd1—C1	131.42 (12)	N2—C13—C12	122.6 (4)
O2—Cd1—C1	28.37 (9)	N2—C13—C14	113.5 (4)
N2—Cd1—C1	100.09 (11)	C12—C13—C14	123.9 (4)
O1—Cd1—C1	28.15 (10)	N1—C14—N3	111.4 (4)
O4^i^—Cd1—C1	96.43 (10)	N1—C14—C13	123.9 (4)
O3^i^—Cd1—C8^i^	28.54 (10)	N3—C14—C13	124.7 (4)
N1—Cd1—C8^i^	96.98 (11)	N1—C15—C20	109.7 (4)
O2—Cd1—C8^i^	124.94 (11)	N1—C15—C16	129.9 (4)
N2—Cd1—C8^i^	140.36 (12)	C20—C15—C16	120.4 (4)
O1—Cd1—C8^i^	101.92 (10)	C17—C16—C15	116.6 (4)
O4^i^—Cd1—C8^i^	28.26 (9)	C17—C16—H16	121.7
C1—Cd1—C8^i^	114.01 (12)	C15—C16—H16	121.7
O1—C1—O2	122.0 (4)	C16—C17—C18	122.7 (4)
O1—C1—C2	119.7 (4)	C16—C17—H17	118.7
O2—C1—C2	118.2 (3)	C18—C17—H17	118.7
O1—C1—Cd1	61.8 (2)	C19—C18—C17	121.0 (5)
O2—C1—Cd1	60.87 (19)	C19—C18—H18	119.5
C2—C1—Cd1	169.4 (3)	C17—C18—H18	119.5
C7—C2—C3	118.9 (4)	C20—C19—C18	116.5 (4)
C7—C2—C1	121.1 (3)	C20—C19—H19	121.7
C3—C2—C1	119.8 (3)	C18—C19—H19	121.7
C4—C3—C2	120.1 (3)	C19—C20—N3	132.7 (4)
C4—C3—H3	120.0	C19—C20—C15	122.8 (4)
C2—C3—H3	120.0	N3—C20—C15	104.5 (4)
C3—C4—C5	120.8 (3)	C14—N1—C15	106.7 (3)
C3—C4—H4	119.6	C14—N1—Cd1	113.2 (3)
C5—C4—H4	119.6	C15—N1—Cd1	140.1 (3)
C4—C5—C6	118.7 (4)	C13—N2—C9	116.8 (4)
C4—C5—C8	119.8 (4)	C13—N2—Cd1	115.5 (3)
C6—C5—C8	121.3 (4)	C9—N2—Cd1	126.6 (3)
C7—C6—C5	120.7 (4)	C14—N3—C20	107.7 (4)
C7—C6—H6	119.6	C14—N3—H1A	134 (4)
C5—C6—H6	119.6	C20—N3—H1A	119 (4)
C6—C7—C2	120.6 (4)	C1—O1—Cd1	90.1 (2)
C6—C7—H7	119.7	C1—O2—Cd1	90.8 (2)
C2—C7—H7	119.7	C8—O3—Cd1^ii^	92.9 (2)
O4—C8—O3	120.8 (4)	C8—O4—Cd1^ii^	89.3 (2)
O4—C8—C5	120.1 (4)		
			
O3^i^—Cd1—C1—O1	38.9 (3)	C20—C15—N1—C14	−0.2 (4)
N1—Cd1—C1—O1	−165.9 (2)	C16—C15—N1—C14	178.1 (4)
O2—Cd1—C1—O1	−171.1 (4)	C20—C15—N1—Cd1	177.6 (3)
N2—Cd1—C1—O1	−90.0 (2)	C16—C15—N1—Cd1	−4.1 (7)
O4^i^—Cd1—C1—O1	92.4 (2)	O3^i^—Cd1—N1—C14	−118.0 (3)
C8^i^—Cd1—C1—O1	69.3 (2)	O2—Cd1—N1—C14	84.9 (3)
O3^i^—Cd1—C1—O2	−150.07 (19)	N2—Cd1—N1—C14	−6.0 (3)
N1—Cd1—C1—O2	5.1 (3)	O1—Cd1—N1—C14	64.6 (4)
N2—Cd1—C1—O2	81.0 (2)	O4^i^—Cd1—N1—C14	−175.0 (3)
O1—Cd1—C1—O2	171.1 (4)	C1—Cd1—N1—C14	82.4 (3)
O4^i^—Cd1—C1—O2	−96.6 (2)	C8^i^—Cd1—N1—C14	−146.7 (3)
C8^i^—Cd1—C1—O2	−119.6 (2)	O3^i^—Cd1—N1—C15	64.3 (4)
O3^i^—Cd1—C1—C2	−62.2 (16)	O2—Cd1—N1—C15	−92.8 (4)
N1—Cd1—C1—C2	93.0 (16)	N2—Cd1—N1—C15	176.3 (4)
O2—Cd1—C1—C2	87.9 (16)	O1—Cd1—N1—C15	−113.1 (4)
N2—Cd1—C1—C2	168.9 (16)	O4^i^—Cd1—N1—C15	7.3 (4)
O1—Cd1—C1—C2	−101.1 (16)	C1—Cd1—N1—C15	−95.3 (4)
O4^i^—Cd1—C1—C2	−8.7 (16)	C8^i^—Cd1—N1—C15	35.6 (4)
C8^i^—Cd1—C1—C2	−31.8 (16)	C12—C13—N2—C9	−0.5 (6)
O1—C1—C2—C7	−16.2 (6)	C14—C13—N2—C9	179.7 (3)
O2—C1—C2—C7	161.1 (4)	C12—C13—N2—Cd1	168.0 (3)
Cd1—C1—C2—C7	79.0 (17)	C14—C13—N2—Cd1	−11.8 (4)
O1—C1—C2—C3	169.3 (4)	C10—C9—N2—C13	−1.2 (6)
O2—C1—C2—C3	−13.4 (5)	C10—C9—N2—Cd1	−168.2 (3)
Cd1—C1—C2—C3	−95.5 (16)	O3^i^—Cd1—N2—C13	103.7 (3)
C7—C2—C3—C4	−1.6 (6)	N1—Cd1—N2—C13	9.9 (3)
C1—C2—C3—C4	173.0 (4)	O2—Cd1—N2—C13	−92.5 (3)
C2—C3—C4—C5	−2.4 (6)	O1—Cd1—N2—C13	−149.1 (3)
C3—C4—C5—C6	5.0 (6)	O4^i^—Cd1—N2—C13	51.2 (5)
C3—C4—C5—C8	−169.8 (4)	C1—Cd1—N2—C13	−120.6 (3)
C4—C5—C6—C7	−3.6 (6)	C8^i^—Cd1—N2—C13	89.8 (3)
C8—C5—C6—C7	171.1 (4)	O3^i^—Cd1—N2—C9	−89.2 (3)
C5—C6—C7—C2	−0.4 (6)	N1—Cd1—N2—C9	177.0 (4)
C3—C2—C7—C6	3.0 (6)	O2—Cd1—N2—C9	74.7 (3)
C1—C2—C7—C6	−171.6 (3)	O1—Cd1—N2—C9	18.1 (4)
C4—C5—C8—O4	−16.7 (6)	O4^i^—Cd1—N2—C9	−141.6 (4)
C6—C5—C8—O4	168.7 (4)	C1—Cd1—N2—C9	46.6 (4)
C4—C5—C8—O3	159.5 (4)	C8^i^—Cd1—N2—C9	−103.0 (4)
C6—C5—C8—O3	−15.1 (6)	N1—C14—N3—C20	−0.7 (5)
N2—C9—C10—C11	2.1 (7)	C13—C14—N3—C20	179.3 (4)
C9—C10—C11—C12	−1.3 (7)	C19—C20—N3—C14	−179.2 (4)
C10—C11—C12—C13	−0.2 (6)	C15—C20—N3—C14	0.5 (4)
C11—C12—C13—N2	1.1 (6)	O2—C1—O1—Cd1	−9.2 (4)
C11—C12—C13—C14	−179.1 (4)	C2—C1—O1—Cd1	168.0 (3)
N2—C13—C14—N1	6.8 (6)	O3^i^—Cd1—O1—C1	−148.3 (2)
C12—C13—C14—N1	−173.0 (4)	N1—Cd1—O1—C1	29.0 (4)
N2—C13—C14—N3	−173.2 (4)	O2—Cd1—O1—C1	5.1 (2)
C12—C13—C14—N3	7.0 (6)	N2—Cd1—O1—C1	94.8 (2)
N1—C15—C16—C17	−179.1 (4)	O4^i^—Cd1—O1—C1	−91.0 (2)
C20—C15—C16—C17	−0.9 (6)	C8^i^—Cd1—O1—C1	−119.1 (2)
C15—C16—C17—C18	0.4 (7)	O1—C1—O2—Cd1	9.3 (4)
C16—C17—C18—C19	0.1 (7)	C2—C1—O2—Cd1	−167.9 (3)
C17—C18—C19—C20	0.0 (7)	O3^i^—Cd1—O2—C1	49.3 (3)
C18—C19—C20—N3	179.1 (4)	N1—Cd1—O2—C1	−176.0 (2)
C18—C19—C20—C15	−0.6 (6)	N2—Cd1—O2—C1	−102.6 (2)
N1—C15—C20—C19	179.6 (4)	O1—Cd1—O2—C1	−5.1 (2)
C16—C15—C20—C19	1.1 (6)	O4^i^—Cd1—O2—C1	87.3 (2)
N1—C15—C20—N3	−0.2 (4)	C8^i^—Cd1—O2—C1	75.6 (3)
C16—C15—C20—N3	−178.7 (4)	O4—C8—O3—Cd1^ii^	5.2 (4)
N3—C14—N1—C15	0.6 (4)	C5—C8—O3—Cd1^ii^	−171.0 (3)
C13—C14—N1—C15	−179.4 (3)	O3—C8—O4—Cd1^ii^	−5.0 (4)
N3—C14—N1—Cd1	−177.9 (2)	C5—C8—O4—Cd1^ii^	171.2 (3)
C13—C14—N1—Cd1	2.1 (5)		

Symmetry codes: (i) *x*+1, −*y*+1/2, *z*+1/2; (ii) *x*−1, −*y*+1/2, *z*−1/2.

### Hydrogen-bond geometry (Å, °)

**Table:**

*D*—H···*A*	*D*—H	H···*A*	*D*···*A*	*D*—H···*A*
N3—H1A···O2^iii^	0.83 (2)	2.17 (3)	2.882 (5)	144 (4)
N3—H1A···O3^iv^	0.83 (2)	2.46 (4)	2.988 (5)	123 (4)

Symmetry codes: (iii) −*x*+1, −*y*, −*z*+1; (iv) −*x*+1, *y*−1/2, −*z*+1/2.
